# Surgical Management of a Mild Case of Frontonasal Dysplasia: A Case Report and Review of Literature

**DOI:** 10.7759/cureus.12821

**Published:** 2021-01-20

**Authors:** Bar Y Ainuz, Erin M Wolfe, S. Anthony Wolfe

**Affiliations:** 1 Plastic and Reconstructive Surgery, Florida International University, Herbert Wertheim College of Medicine, Miami, USA; 2 Plastic and Reconstructive Surgery, University of Miami Miller School of Medicine, Miami, USA; 3 Plastic and Reconstructive Surgery, Nicklaus Childrens Hospital, Miami, USA

**Keywords:** frontonasal dysplasia, frontonasal dysostosis, median cleft face syndrome, plastic and reconstructive surgery, surgical case report

## Abstract

Frontonasal dysplasia (FND) is a rare congenital craniofacial cleft syndrome associated with a spectrum of midline facial bone and soft-tissue malformations. When present, the physical features of FND are often obvious and classified at birth. The resultant facial deformities have the potential to negatively influence psychosocial health and quality of life. Reconstructive surgical intervention in early childhood can serve to restore facial contour and alleviate psychological stress. In this report, a case of a 14-year-old female with previously undiagnosed mild form of FND presented for reconstructive surgery evaluation and underwent several procedures including sliding advanced genioplasty, submucosal resection of the nasal turbinates, open rhinoplasty, and bilateral transnasal medial canthopexies. The patient had subsequent nasal tip recontouring for persistent supratip fullness. The patient achieved an acceptable esthetic outcome and was satisfied with her physical appearance. This case emphasizes the subtle presentation and reconstructive surgical options of a mild case of FND that was diagnosed at a later age, unlike the more severe phenotypes of the syndrome and other common craniofacial anomalies that are usually diagnosed and treated in early childhood. Multidisciplinary craniofacial care teams should be able to correctly diagnose and implement the appropriate surgical interventions in patients with milder forms of FND.

## Introduction

Frontonasal dysplasia (FND) is a rare congenital orofacial cleft syndrome of early embryogenic development resulting in a broad phenotypic presentation of midline head and face malformations [1,2]. Patients with FND characteristically have at least two of the following features: hypertelorism; midline nasal cleft or facial groove; broad nasal bridge and/or nasal tip deformity; central hair pattern such as a widow’s peak; and median cleft lip and palate [3]. However, the severity of the manifestations is widely variable and may include other syndromic findings such as cranium bifidum occultum, craniosynostosis, agenesis of the corpus callosum, mental retardation, small or missing eyes, auricular anomalies, dentoskeletal abnormalities, structural cardiovascular malformations, and genital abnormalities [2-6]. Of these signs, perhaps the most recognized is the nasal cleft, which can range from mild broadening of the tip to nasal bifidity and may lead to narrowing of the nostrils causing airway obstruction and breathing difficulty [3,6,7]. 

As the classification of craniofacial clefts changed over the years, FND adopted several names including Tessier cleft no. 0, frontonasal dysostosis, median cleft face syndrome, median facial dysplasia, and most recently median craniofacial hyperplasia [8,9]. There is currently no consensus on the preferred method for FND classification [10]. Notably, features of FND may be seen as part of broader syndromes associated with a constellation of anomalies not seen in isolated FND such as frontofacionasal and craniofrontonasal syndromes, which include further ocular and musculoskeletal anomalies, respectively [4,11].

Although the mode of inheritance of FND is not definitive, most cases are sporadic, with familial and multifactorial patterns reported in the literature [2]. FND is usually diagnosed clinically by its obvious physical features detected on prenatal ultrasound or physical examination at birth [10]. However, genetic testing may be beneficial as the phenotypic variability and mode of inheritance partly depend on the genetic classification; type 1 FND secondary to ALX3 gene mutation, type 2 FND secondary to ALX4 gene mutation, and type 3 FND secondary to ALX1 gene mutation [2-4]. Within each type of FND, the severity of the phenotype may range from mild to severe [11]. 

Literature describing the reconstructive surgical management of FND is limited and encompasses various multistaged bony and soft-tissue interventions tailored to the craniofacial features expressed in each individual [3,7,12]. These include invasive facial bipartition surgery for correction of hypertelorism, specialized rhinoplasty techniques for correction of the nasal deformity, cleft lip and palate repair, laser therapy for abnormal hair growth, orthognathic intervention to treat malocclusion, and bone and fat grafting for facial recontouring to deficient areas of the face associated with the midline facial groove [3,4,6,7,10,12-18]. 

These operations, among other surgical options for associated defects, are generally performed in prepubescent children beginning in infancy or early childhood to restore normal function and anatomy of the aforementioned disfigurements, as well as mitigate social stigmatization during critical childhood development [3,12,19]. Most patients diagnosed with FND present with classic features at a young age; thus, it is unusual for patients with FND to go through their childhood undiagnosed and/or untreated. This report describes the presentation of the characteristic features and respective surgical interventions implicated in a case of untreated mild FND in an adolescent female.

## Case presentation

**Figure 1:**
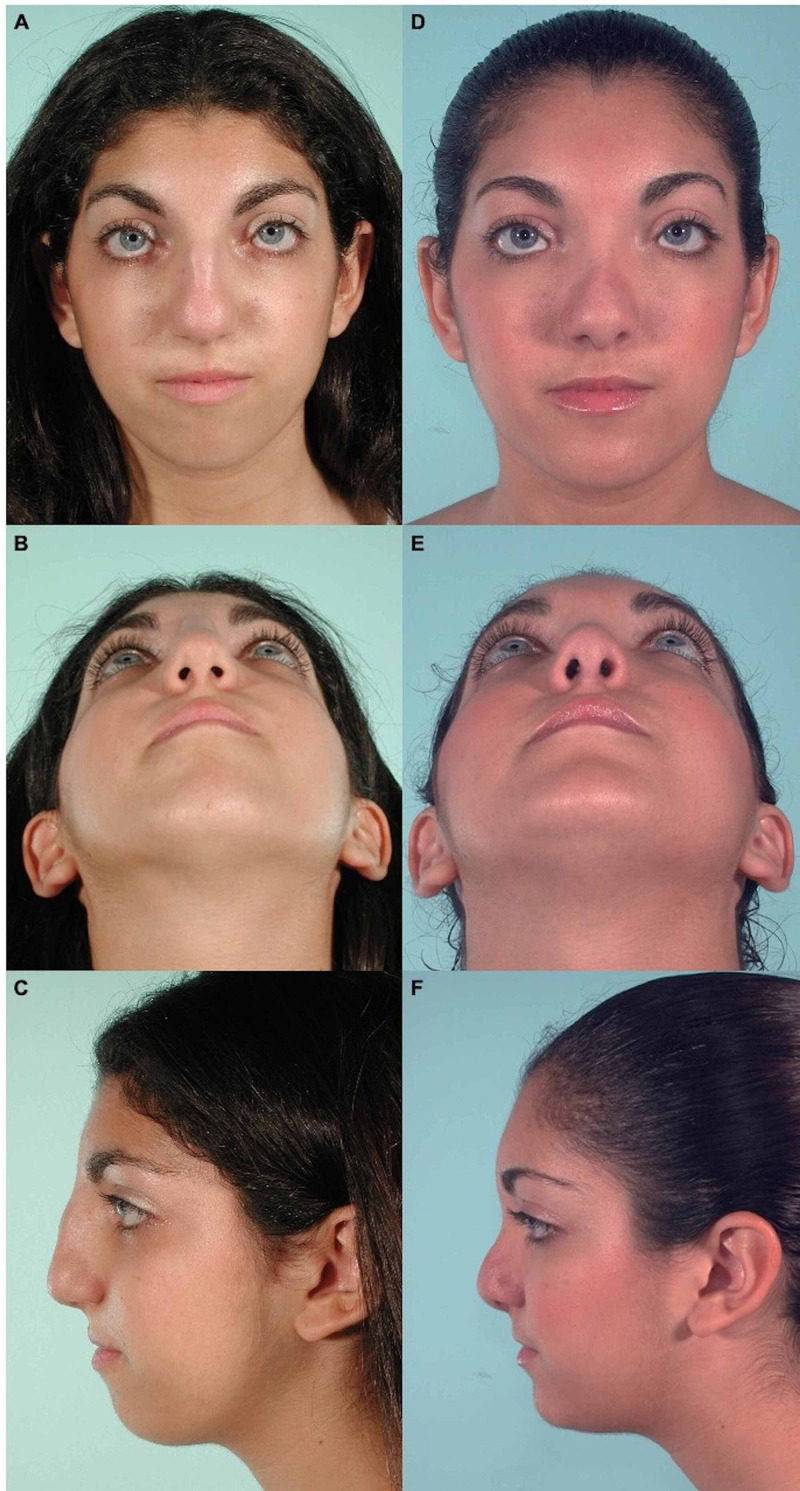
Preoperative and postoperative images of a 14-year-old female with frontonasal dysplasia comparing the esthetic outcomes following sliding genioplasty, open rhinoplasty, submucosal resection of nasal turbinates, bilateral medial canthopexies, and nasal tip recontouring. A. Preoperative frontal view. B. Preoperative basilar view. C. Preoperative profile view. D. Postoperative frontal view. E. Postoperative basilar view. F. Postoperative profile view.

A 14-year-old female presented to the outpatient pediatric reconstructive surgery clinic for evaluation and correction of a previously undiagnosed syndromic congenital facial malformation. The patient did not have a history of developmental delay or family history of syndromic diagnoses. On physical examination the patient had characteristic features classified by the senior author as FND including a low forehead with a central hair pattern resembling a widow’s peak, mild telecanthus, substantial retrogenia, and profound nasal deformity. The patient showed a very large upper dorsal kyphosis extending well out in front of her forehead, shortened columella, and a very broad almost bifid nasal tip with extremely narrow nostrils causing airflow obstruction (Figure 1A-1C). The patient previously had orthodontic treatment for malocclusion with excellent results, but began experiencing lip seal problems and visible strain of her mentalis muscle after treatment. No genetic testing was performed during the patient’s clinical course. The patient and her family opted for surgical restoration of the deformity.

The senior author began with a subperiosteal dissection of the mandibular symphysis through a lower buccal incision in preparation for a sliding genioplasty. Horizontal osteotomies were performed above the lower border of the mandible and below the mental nerves to free the advanced segment, which was advanced anteriorly 6.5 mm to increase the projection of the chin. Next, a coronal incision was made and dissection was carried out to the supraorbital ridges and down to the medial canthal tendons where the dorsal kyphosis was visualized coming off of the nose at a higher level than the forehead. Due to the small nostril size, access to the nasal elements for an open rhinoplasty was made via a columellar base incision. The nasal kyphosis was removed en bloc, leaving a large flattened area on the dorsum of the nose otherwise called an open roof deformity which was closed with infractures. The previously harvested nasal cartilage was used to recontour the columellar strut in order to increase the structural integrity of the tip and provide a scaffold on which the nasal tip can be corrected. The boxy nasal tip was reshaped by trimming the lower lateral cartilages and the alar lining was raised with two percutaneous sutures taken through the alar crease. To increase the size of the nasal passages and improve airflow, the surgeon subsequently performed a submucosal resection of the nasal turbinates. Next, the intercanthal distance was measured at 42 mm, and bilateral medial canthopexies were performed with transnasal wires, acting to stabilize the nasal bones as well as tighten and approximate the canthi. There were no intraoperative complications, and the patient was taken to the recovery room in good condition. 

Postoperative follow-up at one month showed significant improvement of her facial, nose, and chin contour. At one year postoperatively, the patient returned to the clinic for concerns of a persistent wide nasal tip. Thus, a nose revision was performed to thin out the nasal tip with no complications. Following the revision, the nasal tip continued to show mild supratip fullness, but the patient was satisfied with her current appearance and did not wish to undergo further surgical intervention (Figure 1D-1F). 

 

## Discussion

Patients with FND may present with a broad constellation of features and of varying severity. However, the pattern of features seen in FND should be appropriately recognized, leading to a correct diagnosis, understanding the underlying pathogenesis, and formulating a sound surgical plan. This case is unique due to the mild form of the syndrome with subtle features, and the late presentation in adolescence without a previous diagnosis. Although this patient did not receive genetic testing, it is hypothesized that she may have had type 1 FND due to this type’s association with milder forms, the presence of characteristic facial features, and absence of a family member with a syndromic diagnosis hinting at a sporadic or an autosomal recessive inheritance pattern [11]. The reconstructive surgical management is generally not affected by the genetic subtype as each structural abnormality is addressed based on its severity and effect on the patient’s quality of life [15,16]. To the best of our knowledge, surgical intervention for FND in older patients has only been reported in two cases of a 16-year-old boy and a 17-year-old girl with obvious severe FND malformations [10,14]. 

The patient’s mild physical deformities were treated with surgical repair using several standard osteoplastic operations, resulting in significant improvement. She was able to achieve a satisfactory result without unnecessary invasive procedures such as facial bipartition for more severe forms of FND exhibiting pronounced orbital hypertelorism [16]. Instead, bilateral medial canthopexies were sufficient at reducing the appearance of her telecanthus. As with the majority of FND cases, the patient’s main concern was her significant nasal deformity with the major features including her prominent kyphosis, shortened columella, broad nasal tip, and constricted nostrils. There is currently no consensus regarding the preferred technique or optimal timing of surgery for correction of the nasal deformities seen in FND [7,12,16,17]. There are sparse reports of young children with mild cases of FND describing positive outcomes following nasal reconstruction corrected with soft-tissue redraping, external rhinoplasty, and standard open rhinoplasty techniques [12,13,16-18]. Soft-tissue manipulation alone is sometimes not enough to address the deformity despite mild presentations. The majority of these cases reported using an open rhinoplasty approach, which provides a wide surgical field while preserving the major vascular supply to the nasal structures, allowing for better manipulation of the nose [16]. Similarly, in this case, the patient’s nasal deformity was sufficiently corrected with open rhinoplasty and complimentary chin augmentation to balance the proportion of nose protuberance, as well as resection of her nasal turbinates to improve nasal airflow. 

As implied by this case, patients with mild presentations of the syndrome may potentially go undiagnosed at an early age until the facial malformations become socially apparent, driving the patient to seek reconstructive evaluation for their facial esthetics. Multidisciplinary craniofacial care teams should be aware of milder forms of FND as the manifestations can be more subtle and present at a later age, but also lead to psychological morbidity due to social stigmatization and self-perception in affected individuals [12,20]. 

## Conclusions

FND is a rare orofacial cleft syndrome of varying severity. The resultant facial deformities may lead to psychological morbidity in affected individuals and may present as subtle abnormalities at older ages. This case discusses the clinical indications and surgical management of a rarely encountered mild case of frontonasal dysplasia in an adolescent female.
